# Diet quality and environmental impact of university students’ food choices at a South African university

**DOI:** 10.3389/fnut.2025.1668622

**Published:** 2025-10-15

**Authors:** Sanrika Sahadeo, Ashika Naicker, Onwaba Makanjana, Oluwasiji O. Olaitan

**Affiliations:** Department of Food and Nutrition Consumer Sciences, Durban University of Technology, Durban, South Africa

**Keywords:** diet quality, nutrient adequacy, 24-h food recall, Global Diet Quality Score, sustainability, environmental impact, carbon footprint, university students

## Abstract

**Background:**

South Africa faces a triple burden of malnutrition. The country’s food system, characterized by high consumption of resource-intensive animal and processed foods, contributes significantly to greenhouse gas emissions. Global policy frameworks increasingly emphasize sustainable diets, but national strategies and implementation efforts in South Africa are still emerging. The climate emergency has amplified global efforts to promote sustainability, yet the environmental impact of dietary choices remains underexplored in South Africa. This study examines diet quality and the environmental impact of food choices of students in a South African university, offering insights into sustainability from a young consumer perspective.

**Methods:**

Dietary data were collected using a repeated 24-h food recall method over two non-consecutive days, including a weekend day, for 400 students. Nutrient adequacy was assessed using the cut-off points of recommended daily allowance (RDA) and adequate intake, while diet quality and non-communicable disease (NCD) risk were evaluated using the Global Diet Quality Score (GDQS). The environmental impact of students’ diets was estimated using the Plate Up for the Planet carbon footprint calculator.

**Results:**

Male students exceeded carbohydrate (310.8 g) and protein (88.06 g) requirements but had notable micronutrient deficiencies, including calcium (74.3% prevalence of inadequacy [POI]), magnesium (96.7% POI), and vitamin D (92.8% POI). Female students showed deficiencies in dietary fiber, calcium (94.0% POI), and folate (92.3% POI). The GDQS revealed that 99.8% of students (37.8% males, 62.0% females) were at high risk for nutrient inadequacy and non-communicable diseases (NCDs). The mean carbon footprint analysis showed that male students (5671.55CO₂e) had a higher environmental impact than females (5020.36 CO₂e).

**Conclusion:**

Nutrient inadequacy and poor diet quality are highly prevalent among university students, predisposing them to a high risk of NCDs and contributing to a high amount of greenhouse gas emissions. University food environments significantly influence student diets, necessitating policy interventions to promote sustainable food choices while reducing environmental impact.

## 1 Introduction

Sustainable diets, as defined by Burlingame and Dernini, are diets with low environmental impact and contribute to food and nutrition security and a healthy life for both the present and future generations (1). Diet and nutrition status can significantly influence the risk of non-communicable diseases (NCDs), including obesity, diabetes, cardiovascular disease, and cancer (2).

Therefore, consumers need to consume diets that are predominantly plant-based and low in salt, saturated fats and added refined sugars, which is recommended as part of a healthy lifestyle to mitigate both health and environmental challenges (3). These diets are often associated with reduced risks of NCDs and a decreased risk of early mortality. This recommendation supports the body of research indicating that consuming less processed meat (such as sausages and cured, smoked, and salted meats) and red meat may aid in preventing several NCDs.

However, stringent plant-based diets, particularly vegan diets, also raise concerns about micronutrient deficiencies such as iron and vitamin B12 (4). These diets have a lower environmental impact than meat and dairy products. For that reason, to create a climate that supports nutritious, healthy diets, policymakers must develop and implement well-targeted policy interventions (2).

For consumers to adopt more environmentally friendly food choices which increase the effectiveness of public health and food policy activities, authorities need to disseminate information on sustainability issues and the environmental impact of dietary patterns of the population (5). However, limited studies have been conducted in South Africa surrounding food and nutrition sustainability, especially among young adults such as university students (6, 7). Food consumption preferences in South Africa have evolved significantly during the last few decades, and projections point to a substantial change in the coming decades. There has been a noticeable increase in the consumption of processed and convenience foods, animal-source foods, and sugary beverages, especially in urban settings (8, 9). Rural households are also becoming more dependent on processed foods from the established retail sector because of insufficient agricultural extension services (10, 11). Consequently, this nutrition transition is increasing the marginalization of traditional diets which are nutritious and sustainable to meet the nutritional needs of the growing populations of South Africans, and to address the current food insecurity to intensified by climate change and inflation in the country’s economy. Alarmingly, the current younger generation is increasingly losing the ability to identify indigenous foods and traditional food processing techniques, a trend largely driven by the proliferation of convenience and fast foods, as well as increased urbanization (12, 13). Furthermore, South Africa has undergone a significant nutrition transition over the past 20 years, driven by an evolving food system. This has contributed to the prevalence of obesity and associated complications, as well as micronutrient inadequacies among young adults as highlighted by findings from the African PREDICT study and national dietary surveys (14). In the African PREDICT cohort, over 70% of participants did not meet the Estimated Average Requirements (EARs) for 17 out of the 19 assessed micronutrients (15). More than half of the cohort had intakes below the EARs for essential nutrients including calcium, magnesium, folate, pantothenic acid, biotin, and vitamins A, C, D, and E (15). These deficiencies persist despite national fortification policies, suggesting a continued gap in dietary quality, particularly concerning vitamin A and folic acid (13). Should the current nutrition situation persist without intervention, the prevalence rate of non communicable diseases (diabetes, cardiovascular diseases and cancer) will be accelerated among young adults. This would endanger the country’s economically active population and impose substantial economic burdens due to escalating healthcare expenditure and decreased workforce productivity.

However, parts of the factors affecting the diet quality of many young South African adults include ethnic and socioeconomic disparities, which exacerbate the problem of micronutrient deficiencies (particularly for calcium, magnesium, folate, biotin, and vitamins A, C, D, and E) among young adults in South Africa (15). Individuals identifying as Black and those from lower socioeconomic status (SES) backgrounds demonstrate significantly lower intakes of several essential micronutrients compared to their White and higher SES counterparts (15). For example, calcium and potassium intake among Black participants was substantially lower, with up to 80% not meeting recommended levels, likely reflecting a dietary reliance on energy-dense, nutrient-poor staple foods (16, 17).

In the study conducted by Sahadeo et al. among university students in South Africa, a complex relationship between food choices, sustainability knowledge, and perceived barriers and drivers was established. While some of the university students demonstrated a strong understanding of healthy and sustainable diet, a significant number of them were not aware of key concepts related to environmental impact, such as product environmental footprint (65.2%), life-cycle assessment (66.2%), food miles (58.5%), and greenwashing (64.9%). The gaps suggest that although students may be motivated to make healthier food choices, they often lack the knowledge to assess the broader environmental impacts of their diets (18).

In South Africa, climate change goals seem to have taken precedence over policy initiatives to support sustainable diets. There is little information available that is known about the environmental effects of diet and food choices. The 27th Conference of the Parties (COP27), acknowledged the interlinkages between climate change, food systems and health. It called for integrated policies that align environmental sustainability with improved nutrition outcomes (19). Additionally, the COP28 marked a breakthrough by elevating food systems in climate discourse, integrating them into key agenda items and generating commitments and finance, though gaps remained in targeting emissions and smallholder inclusivity (20). However, COP29 maintained many COP28 gains but fell short on major new commitments or funding increases for food systems (21). Although COP29 made limited progress in advancing the groundwork established at COP28 and fell short of achieving significant commitments regarding the pivotal role of food systems in climate action. In Baku, a more coordinated and motivated food systems community worked collaboratively to sustain forward momentum. Nonetheless, substantial work remains in the lead-up to COP30, and this community will be essential in driving continued progress (21).

In recent years, the SDGs have placed considerable focus on evaluating the carbon emissions associated with different foods and food-related products. Changing eating behaviors across various countries presents significant challenges, particularly given the growing awareness about carbon footprints and the phenomenon of global warming (22). The carbon footprint associated with dietary patterns plays a critical role in influencing global GHG emissions, with food production responsible for an estimated one-quarter of total emissions (23). Animal-derived food products, particularly beef and dairy, represent some of the most significant sources of methane emissions stemming from livestock and manure management practices (24). For example, 1 kg of beef produces approximately 22 kg of CO2e emissions, whereas pork generates roughly 6 kg, and chicken emits only about 1.5 kg (24). Conversely, plant-based foods, which include grains, legumes, and vegetables, typically exhibit a significantly lower carbon footprint, making them preferable sustainable alternatives (25).

In filling this gap, this study explored sustainability from the young adult consumers’ perspective to advance transformative strategies for sustainability, climate change goals, and enhanced health toward nature-positive solutions. This study contributes to the knowledge gap by assessing the diet quality and carbon footprint of university students at the Durban University of Technology (DUT). The aim is to inform future strategies to align student diets with the Sustainable Development Goals (SDG 2030) and Africa’s Agenda 2063.

## 2 Methods

### 2.1 Study design, population and sampling

This study included 400 undergraduate and postgraduate students aged 18–34 years from the Durban University of Technology’s (DUT’s) Steve Biko, Ritson, and ML Sultan campuses. Durban University of Technology is a multi-campus university which is situated in the province of KwaZulu-Natal, South Africa, and focuses on higher education, technological training, research, and innovation. The DUT encompasses six faculties (Accounting & Informatics, Applied Sciences, Arts and Design, Engineering and Built Environment, Health Sciences and Management Sciences) with a population of approximately 33,000 students (26). According to Taherdoost, a minimum sample size of 385 was calculated (with a 5% precision and 95% confidence interval) based on 17, 840, 000 youth population (18–34 years) in South Africa (27, 28). Respondents were recruited through convenience sampling at key hub locations across all three campuses (Steve Biko, Ritson, and ML Sultan campuses). The inclusion criteria for this study required that all students be between 18 and 34 years old and registered as either undergraduate or postgraduate students. Students were selected from key hub areas, specifically the Steve Biko Campus, Ritson Campus, and ML Sultan Campus. The study included both male and female students, and ensured representation from a wide array of ethnic backgrounds. Additionally, individuals of all abilities, encompassing both abled and disabled persons, were included to ensure a diverse and representative sample. The exclusion criteria for this study comprised of individuals younger than 18 years or older than 34 years of age, all DUT staff, outsourced general workers, maintenance workers, and security guards.

### 2.2 Dietary assessment

A repeated 24-h food recall was conducted on two non-consecutive days, including a weekday and a weekend day, resulting in two recalls per student. Thereafter, the mean of the dietary intake was calculated and used for further analysis. The multiple-pass method, using standard food models and household measurement tools to enhance recall accuracy, was employed using trained fieldworkers. Inter-rater reliability was assessed for accuracy in data collection among field workers during the training. Data collection adhered to protocols validated in prior South African nutrition studies (29). The information obtained from the 24-h food recall included the type of food consumed, portion sizes, preparation methods and ingredients used.

Portion sizes were determined using measuring cups, measuring bowls, plates of different sizes, cups, mugs, glasses, measuring spoons, dishing spoons and food aid props. The first step of the 24-h food recall was to determine the time of day when the respondents consumed the food. The respondents were then asked to list the foods that they consumed at specific times. Thereafter, respondents had to describe the preparation methods used to cook the meals. A dietary toolkit guided respondents in estimating portion sizes. Respondents had to indicate if this was their usual diet that they followed. The 24-h food recall was conducted using consistent methods (29). A time commitment of 20–30 min was required per 24-h recall. Data collection commenced from 24 July 2023 to 10 December 2023 and a total of 400 repeated 24-h food recalls were collected from this study. This study received ethical approval from the Institutional Research Ethics Committee (IREC 102/23), and written informed consent was obtained from all student participants prior to data collection.

### 2.3 Dietary analysis

The repeated 24-h food recall data were captured onto the FoodFinder version 3 software program of the South African Medical Research Council to determine the nutrients consumed over the two non-consecutive days. The foods that each respondent consumed each day were recorded, and a report containing an average daily nutrient intake of the 2 days was generated. Data from the FoodFinder was exported to Microsoft Excel, and the mean daily nutrient intakes and standard deviations were calculated. Of the four Dietary Reference Intakes (DRI), the Estimated Average Requirement (EAR) and Adequate Intake (AI) were used to assess the nutrient intake (29). The EAR was selected as the recommended DRI for assessing the nutritional status of population groups, defined by demographic profiles, including age, gender and lifecycle stage (30). The AI is a recommended average daily nutrient intake level, based on experimentally derived intake levels or approximations of observed mean nutrient intake by a group (or groups) of apparently healthy people that are assumed to be adequate. The AI values were utilized for nutrients that lack a defined EAR (31). The EAR is defined as the quantity of a nutrient that is thought to be sufficient for 50% of the population of a certain demographic group. The proportion of EAR was calculated using the mean intake value of each nutrient with its corresponding EAR. Subsequently, the incidence of inadequate intake was ascertained through the application of the EAR cut-point method. This method applies to most nutrients, except for energy (31, 32). Minimum recommended daily calorie intake of 9,204.8KJ and 7,531.2KJ for healthy, moderately active male and female adults was used as a cut-off point, respectively. The mean of nutrient intake was compared to EAR/AI cut off points and the incidence of inadequate intake was ascertained. This method applies to most nutrients, except for energy (31, 32). The AI values were utilized for nutrients that lack a defined EAR (33). The AI is predicted on either observed or experimentally derived estimates of the average nutrient consumption of a healthy demographic group. It is postulated that exceeding the AI for nutrient intake indicates a reduced risk of inadequate intake (34).

### 2.4 Overview of the Global Diet Quality Score

To determine the dimensions of diet quality for nutrient adequacy and NCD risk, the Global Diet Quality Score (GDQS) was used (35). The GDQS represents a comprehensive metric meticulously developed to evaluate dietary quality and compliance with nutritional guidelines across diverse populations globally (36). Developed by the World Health Organization in collaboration with researchers from various institutions, the GDQS encompasses a wide range of dietary elements, including the consumption of fruits, vegetables, whole grains, legumes, nuts, and beneficial fats, while simultaneously restricting the intake of sugars, saturated fats, and ultra-processed foods (37).

The GDQS is calculated based on the consumption of 25 food groups for the period of twenty-four hours. The reference food groups are categorized into 16 healthy food groups, 7 unhealthy food groups, and 2 other unhealthy food groups (red meat, high-fat dairy) when consumed in excessive amounts. The GDQS is classified into high (<15 points), moderate (≥15 and <23 points) and low (≥23 points) risk of nutrient inadequacy and NCDs (37). The 16 healthy food groups include: (1) dark green leafy vegetables, (2) cruciferous vegetables, (3) deep orange vegetables, (4) other vitamin A–rich fruits and vegetables, (5) citrus fruits, (6) other fruits, (7) legumes, (8) nuts and seeds, (9) whole grains, (10) eggs, (11) fish and shellfish, (12) poultry, (13) fermented milk products, (14) liquid oils, (15) low-fat dairy, and (16) other vegetables. The 7 unhealthy food groups consist of: (1) sugar-sweetened beverages, (2) sweets and desserts, (3) refined grains and baked goods made with refined flour, (4) white roots and tubers prepared in unhealthy ways (e.g., deep-fried), (5) processed meats, (6) packaged salty snacks, and (7) fast food and deep-fried foods. These classifications reflect the potential of each food group to either contribute to or detract from overall diet quality, nutrient adequacy, and long-term health outcomes.

The GDQS is a valuable tool for policy-making and public health monitoring since it allows for assessing how dietary patterns influence health outcomes such as obesity and NCDs. Recent studies have shown that higher GDQS scores are associated with improved health, highlighting the need for global initiatives to promote healthier eating habits (35). Given the increased prevalence of diseases related to diets in many countries, the GDQS offers vital information to formulate nutrition policies and initiatives appropriate for national dietary customs and cultural backgrounds.

### 2.5 Assessing the environmental footprint of dietary choices

Carbon footprint analysis was conducted using the Plate Up for the Planet calculator, estimating diet-related CO₂ emissions based on reported intake (38). The Plate Up for the Planet carbon calculator (PUPCC) assists individuals by evaluating the environmental consequences of their dietary selections, with particular emphasis on carbon emissions (39). According to the United Nations, carbon footprint refers to the total greenhouse gas emissions which include carbon dioxide, methane and nitrous oxide, converted into a single potential warming effect of carbon dioxide (CO₂). Therefore, carbon footprint is measured in kilogram of carbon dioxide equivalents (kgCO₂e) (40). It offers valuable insights into the extent to which various food items contribute to an individual’s carbon footprint and promotes the adoption of more sustainable dietary practices (41).

The PUPCC has numerous advantages, including raising awareness regarding the carbon footprint associated with dietary selections, the provision of an accessible, user-friendly interface for engagement, and the facilitation of data collection pertinent to research on food-related emissions. However, disadvantages such as potential inaccuracies stemming from underlying assumptions within the model, a restricted emphasis on dietary selections without accounting for broader environmental considerations, and a dependence on self-reported data may exhibit inconsistencies. The PUPCC did not include certain food items. In such cases, the most comparable available food item was selected as a substitute to estimate the associated carbon footprint. While these limitations are acknowledged, efforts were made to mitigate them by applying consistent substitution criteria, choosing conservative estimates where applicable and clearly documenting all assumptions to promote transparency and enhance the reliability of the findings. Food quantities were entered in grams into the carbon calculator, and the 24-h dietary recall data were submitted directly to the website and expressed in kilograms per day. Data from the second day were excluded from the analysis, as the second 24-h food recall was conducted telephonically, which may have introduced inconsistencies in portion size estimation.

### 2.6 Statistical analysis

Statistical Package for Social Sciences version 29.0 was used for descriptive and inferential statistics, such as the Mann–Whitney U test and the Chi-square test. The GDQS and food carbon footprint data were analysed using the one-sample t-test to calculate the mean, median, standard deviation, and confidence interval. Additionally, the Mann–Whitney U test was employed to compare the mean ranks of the carbon footprint across genders, as the data were not normally distributed.

## 3 Results

### 3.1 Sociodemographic characteristics of respondents

Table 1 presents sociodemographic information of respondents. More than half (*n* = 248, 62%) of the respondents were females, while 38.0% (*n* = 152) were males. A large proportion (98.3%, *n* = 393) were within the age of 18 to 26 years and 84.7% (*n* = 339) were Blacks while 42.0% (*n* = 168) were first-year students, 31.0% (*n* = 124) were in second year, 20.8% (*n* = 83) were in third year, 4.0% (*n* = 16) were in fourth year, while 2.2% (*n* = 9) were postgraduate students.

**Table 1:** Sociodemographic information of respondents.

Characteristics	Frequency (*n* = 400)	Percentage (100%)
Gender		
Male	152	38
Female	248	62
Age (years)		
18–26	393	98.3
27–34	7	1.7
Race		
Black	339	84.7
White	2	0.5
Indian	49	12.3
Colored	10	2.5
Level of Study		
First year	168	42
Second year	124	31
Third year	83	20.8
Fourth year	16	4
Postgraduate	9	2.2

### 3.2 Dietary information of respondents

Tables 2, 3 represent the results of the first and second 24-h food recalls for men and women, respectively. Table 2 notes the key observations for energy and nutrient intake for men. The prevalence of inadequate intake was calculated using the cut-point method, highlighting specific nutrient deficits among the group. While the mean energy intake (9,691 kJ) exceeded the EAR (9,205 kJ), nearly 59.9% of respondents failed to meet the energy requirement, suggesting an uneven energy distribution among men in this study. Total fat intake (mean: 77.5 g) was slightly below the EAR (86 g), and 75.7% of respondents consumed inadequate amounts. The mean intake for dietary fiber (26.69 g) was substantially below the AI of 38 g, with 94.7% of respondents having inadequate fiber intake. The following key observations were made for micronutrient deficiencies:

**Table 2:** Nutrient intake, mean, percentiles and prevalence of inadequate intake of male respondents (*n* = 152).

Nutrient/Calorie	EAR/AI* cut-off points	Mean (SE)	Percentiles					Prevalence of inadequate intake (%)
			10%	25%	50%	75%	90%	
Energy (kJ)	9,205	9691.25 (701.63)	5842.00	7076.75	8676.25	**9909.38**	12106.00	59.9
Total protein (g)	56	88.06 (6.82)	48.12	**59.75**	75.48	93.04	129.14	19.1
Carbohydrate (g)	130	3,108,161 (29.19)	**167.07**	215.34	265.78	343.54	401.35	3.3
Total fat (g)	86	77.5(6.88)	38.17	47.73	65.35	84.90	**105.69**	75.7
Dietary fiber (g)	38*	26.69 (5.55)	10.72	14.60	18.23	26.08	32.73	94.7
Ca (mg)	800*	681.98 (52.52)	275.50	412.50	605.00	**812.88**	1072.90	74.3
Fe (mg)	8	17.98 (1.709)	**8.43**	11.01	14.33	19.00	28.36	7.9
Se (μg)	55	74.72 (7.83)	22.97	36.71	**59.08**	86.50	128.68	47.4
Zn (mg)	11	15.42 (3.03)	6.60	8.33	**11.09**	15.09	1960	48.7
Mg (mg)	420	252.79 (38.18)	101.40	137.13	188.25	254.88	331.45	96.7
P (mg)	700	978.90 (103.42)	491.65	623.25	**817.00**	1021.13	1358.95	34.2
Vitamin A (μg)	900	967.06 (121.16)	207.65	338.63	619.75	**916.75**	1925.50	74.0
Vitamin C (mg)	90	117.96 (7.96)	20.10	46.13	88.75	153.75	268.05	50.7
Vitamin D (μg)	15*	6.89 (0.51)	0.75	2.54	5.78	9.64	13.78	92.8
Vitamin E(μg)	15	17.85 (1.46)	5.31	8.78	13.81	22.79	34.88	55.9
Vitamin K (μg)	120*	61.21 (5.42)	17.11	29.02	39.98	67.04	**129.41**	89.5
Riboflavin	1.3	1.60 (0.11)	0.69	0.94	1.25	1.75	2.78	53.9
Folate (μg)	400	117.01 (20.50)	1.59	14.38	46.13	117.86	302.37	94.1
Niacin	16	26.37 (1.34)	12.03	**17.85**	22.65	30.18	44.62	17.1
Vitamin B6 (μg)	1.1	2.29 (0.29)	0.79	**1.14**	1.71	2.25	3.25	22.7
Vitamin B12 (μg)	2.4	3.81 (0.37)	0.90	1.55	**2.75**	4.25	7.02	41.4
Thiamine (mg)	1.2	1.68 (0.14)	0.78	0.98	**1.39**	1.84	2.69	37.7
Total sugar (g)	166	65.96 (10.79)	20.72	37.93	49.90	69.89	95.17	99.3
Added sugar (g)	36	10.06 (0.98)	0.00	0.00	5.48	16.80	29.76	94.7
Saturated fat (g)	29	23.11 (2.83)	9.39	13.35	18.66	24.39	**33.35**	82.9
Monounsaturated fat (g)	31	24.94 (2.63)	11.01	14.59	21.24	27.49	**33.9**	84.9
Polyunsaturated fat (g)	23	23.04 (1.56)	8.64	12.39	18.10	**29.08**	40.02	66.4
Trans fat (g)	2	1.09 (0.13)	0.09	0.19	0.62	1.21	**2.743**	86.8
Cholesterol (mg)	300	240.49 (16.68)	61.80	101.50	180.00	**317.38**	522.25	73.7

*AI = Adequate intake (33, 65); EAR, Estimated Adequacy Requirement, SE, Standard error. Figures in bold type refer to the level where the EAR/AI is met.

**Table 3:** Nutrient intake, mean, percentiles and prevalence of inadequate intake of female respondents (*n* = 248).

Nutrient/Calorie	EAR/AI cut-off points	Mean (SE)	Percentiles					Prevalence of inadequate intake
			10%	25%	50%	75%	90%	
Energy (KJ)	7,531	7766.78 (159.97)	5182.55	6188.03	7253.50	**9201.38**	10784.80	53.6
Total protein (g)	46	67.15 (1.66)	39.68	**50.18**	64.30	78.84	101.43	19.8
Carbohydrate (g)	130	241.35 (5.46)	**149.53**	181.30	229.43	282.30	340.57	4.4
Total fat (g)	69	32.59 (1.20)	13.25	19.50	28.50	40.86	56.89	95.2
Dietary fiber (g)	25*	17.744 (0.56)	9.79	12.45	16.08	21.16	**26.37**	86.7
Ca (mg)	900*	517.71 (14.59)	257.90	355.38	479.00	624.75	833.25	94.0
Fe (mg)	18	14.91 (0.53)	7.69	9.74	12.85	16.66	**23.68**	79.0
Se (μg)	55	62.26 (2.69)	22.71	37.69	54.78	**74.10**	107.70	50.4
Zn (mg)	8	11.02 (0.37)	5.56	7.519	**9.69**	12.84	17.85	29.8
Mg (mg)	320	181.77 (5.89)	82.45	118.50	168.75	220.88	294.15	93.1
P (mg)	700	757.65 (20.16)	413.75	541.63	**716.25**	894.50	1210.75	48.4
Vitamin A (μg)	700	1308.86 (232.87)	245.60	363.63	571.00	**1042.50**	2405.10	63.3
Vitamin C (mg)	75	121.37 (5.75)	26.50	42.75	**106.25**	177.00	240.90	39.9
Vitamin D (μg)	15*	5.98 (0.35)	1.37	2.46	4.42	7.25	12.67	93.1
Vitamin E(μg)	15	14.34 (0.55)	5.52	8.09	12.44	**18.21**	26.32	64.1
Vitamin K (μg)	90*	71.43 (6.80)	12.62	22.13	39.39	66.00	**148.99**	83.1
Riboflavin	1.1	1.40 (0.06)	0.69	0.86	**1.13**	1.57	2.42	46.0
Folate (μg)	400	121.92 (12.99)	3.15	15.17	50.19	124.19	328.64	92.3
Niacin (mg)	11	21.96 (0.70)	10.29	**14.41**	19.95	26.24	35.87	12.5
Vitamin B6 (μg)	1.1	1.89 (0.06)	0.89	**1.18**	1.67	2.32	3.14	20.6
Vitamin B12 (μg)	2.4	5.78 (0.85)	0.90	1.60	**2.73**	4.33	10.07	42.3
Thiamine (mg)	1.1	1.35 (0.05)	0.71	0.88	**1.18**	1.55	2.14	44.8
Total sugar (g)	132	44.96 (1.67)	16.27	24.49	39.75	61.93	80.44	99.2
Added sugar (g)	24	11.14 (1.05)	0.00	0.20	4.78	14.95	**29.96**	85.1
Saturated fat (g)	23	20.30 (0.59)	10.05	14.06	18.39	**24.93**	34.69	67.3
Monounsaturated fat	26	22.33 (0.69)	9.87	14.89	20.69	**27.98**	36.82	69.4
Polyunsaturated fat	18	20.82 (0.84)	7.67	11.53	17.57	**27.51**	37.26	51.2
Trans fat	2	1.14 (0.09)	0.11	0.28	0.62	1.34	**2.76**	85.1
Cholesterol (mg)	300	230.54 (12.32)	55.90	107.63	173.25	288.50	**505.40**	77.0

*AI = Adequate intake (33, 65); EAR, Estimated Adequacy Requirement, SE, Standard error figures in bold type refer to the level where the EAR is met.

The mean calcium intake was 681.98 mg, 74.3% of respondents failed to meet the AI of 800 mg. The mean intake of magnesium (252.79 mg) was well below the EAR of 420 mg, and 96.7% of respondents exhibited inadequate magnesium intake. The mean intake of vitamin D was 6.89 μg against an AI of 15 μg, with 92.8% of respondents having inadequate vitamin D intake. The mean intake of vitamin K (61.21 μg) is well below the AI (120 μg), with 89.5% of respondents having insufficient intake. The mean intake of folate (117.01 μg) was substantially below the EAR (400 μg), with 94.1% of respondents not meeting the requirement. The notable exceptions were for carbohydrates and protein, in which the average intakes for carbohydrates (310.8 g) and protein (88.06 g) were well above the EARs (130 g and 56 g, respectively).

Table 3 provides data on the nutrient intake of 248 female respondents compared to the EAR or AI guidelines. The prevalence of inadequate intake was calculated using the cut-point method, highlighting specific nutrient deficits among the group. The mean energy intake (7,766 kJ) closely aligns with the EAR (7,531 kJ). However, 53.6% of respondents consumed inadequate amounts, indicating variability in energy intake within the group. Protein intake exceeded the EAR (46 g), with a mean intake of 67.15 g. Only 19.8% of respondents consumed inadequate protein levels, suggesting most women met their protein requirements. The mean carbohydrate intake (241.35 g) significantly exceeded the EAR (130 g), with only 4.4% of respondents showing inadequacy. In contrast, dietary fiber intake (mean: 17.74 g) was significantly below the AI (25 g), with 86.7% of respondents failing to meet the requirement.

Calcium intake was critically low, with a mean intake of 517.71 mg compared to the AI of 900 mg. A notable 94% of respondents consumed insufficient calcium. For iron, while the 90th percentile intake (23.68 mg) exceeded the EAR (18 mg), 79% of respondents had inadequate iron intake, likely reflecting poor dietary diversity. Magnesium intake was also notably low, with a mean of 181.77 mg compared to the EAR of 320 mg, leaving 93.1% of respondents with inadequate intake. Vitamin D intake (mean: 5.98 μg) fell far short of the AI (15 μg), with 93.1% of respondents showing inadequacy. Similarly, vitamin K intake (mean: 71.43 μg) was below the AI of 90 μg, with 83.1% of respondents consuming inadequate levels. The mean folate intake was 121.92 μg, with 92.3% inadequacy among the respondents.

### 3.3 Diet quality and risk of noncommunicable diseases among respondents

Table 4 presents respondents’ global diet quality score categories. The average GDQS of the respondents was 5.5 ± 2.74, with males (5.6 ± 2.79) having a higher score than females (5.4 ± 2.72). The findings indicate that none of the respondents were classified in the low-risk category for nutrient inadequacy and NCDs. Only one respondent was classified as moderate risk, representing 0.3% male and 0.0% female of the total number of respondents. This suggests that a very minimal percentage of respondents are at a moderate level of risk. A significant majority, 99.8% (*n* = 399) respondents, of which there were more females (62.0%, *n* = 248) than males (37.8%, *n* = 151) who were at high risk of NCDs, indicating poor diet quality and potential long-term health risks.

**Table 4:** Respondents’ Global Diet Quality Score categories (*n* = 400).

Characteristics	Male	Female	Total	*F*	*p*
Mean	5.6	5.4	5.5	0.037	0.847
Standard deviation	2.79	2.72	2.74		
Minimum	0	0	0		
Maximum	15.0	14	15.0		
GDQS category					
High risk	151 (37.8%)	248 (62.0%)	399 (99.8%)*		
Moderate risk	1 (0.3%)	0 (0.0%)	0 (0.0%)		
Low risk	0 (0.0%)	0 (0.0%)	0 (0.0%)		
Total	**152 (38.0%)**	**248 (62.0%)**	**400 (100.0%)**		

*GDQS: Global Diet Quality Score. The values in bold indicate the most significant results of the GDQS among males and females and the total sample.

### 3.4 Information on respondents’ carbon footprint

Information on carbon footprint of the respondents is presented in Table 5. The CO₂e is used to group various greenhouse gasses into a single quantity. The amount of CO₂ that would have the equivalent effect on global warming for every quantity and type of greenhouse gas is denoted by the symbol CO₂ (42). The mean carbon footprint for all students was 5267.8 kg CO₂e. Males had a higher mean carbon footprint (5671.6 kg CO₂e) compared to females (5020.4 kg CO₂). The difference in mean is: 5671.6 kg–5020.4 kg = 651.2 kg CO₂e. Males emitted, on average, about 651.2 kg CO₂ more than females. Male students had significantly higher footprints (mean: 5671.6 kg CO₂e) than females (mean: 5020.4 kg CO₂e), *p* = 0.024. This reflects higher meat and processed food consumption among males.

**Table 5:** Respondents’ Carbon footprint for the 24-h food recall and gender-specific carbon footprints.

Characteristics	Male (*n* = 152) (kg CO₂e)	Female (*n* = 248) (kg CO₂e)	Total (*n* = 400) (kg CO₂e)	*Z*	*p*
Mean (SD)	5671.6 (3976.55)	5020.4 (3790.47)	5267.8 (3870.24)	−2.257	0.024
Median (IQR)	4,490 (4,729)	3,795 (4,400)	4,130 (4,700)		
Minimum	880	450	450		
Maximum	30,806	23,910	30,806		

SD: Standard Deviation, IQR: Interquartile Range, kg CO₂e: kilograms of carbon dioxide equivalent.

## 4 Discussion

This study investigated diet quality and its environmental impact among university students, offering insights into sustainable dietary behaviors from a young consumer perspective. The findings are discussed in relation to identified nutritional gaps, health risks, and environmental outcomes, with implications for informing campus food policy in South Africa.

University settings are important in shaping food preferences for several reasons, as they represent a transitional life stage and environment where individuals often develop independent eating habits for the first time (increased autonomy and independence, exposure to diverse food environments, peer and social influences, time and financial constraints) (43). University students display a range of common dietary behaviors, which include the regular consumption of energy-dense snacks, the skipping of meals, particularly breakfast, the excessive intake of processed food while neglecting fruits and vegetables, and tending to opt for convenience foods that require minimal cooking time.

In a study conducted at another university in South Africa, factors such as busy schedules and limited availability of food options on campus caused students to prefer affordable staple foods like bread, rice, maize meal, pasta, eggs, and salty snacks, rather than healthier options such as fruits and vegetables (44). Consequently, these choices often lead students to deviate from sustainable dietary behaviors, as nutritional quality, environmental impact, and long-term health outcomes are frequently overlooked in favor of affordability, accessibility, and taste. These habits, in conjunction with insufficient physical activity and extended engagement with computers and television, may contribute to malnutrition or overnutrition, thereby increasing an individual’s susceptibility to preventable diseases (45).

### 4.1 Nutritional adequacy and diet quality

In this study, nutrient intake indicated that energy and protein were generally adequate for both genders. For the male and female respondents, the prevalence of nutrient inadequacies, especially in dietary fiber, calcium, magnesium, vitamin D, vitamin K, and folate, was noted. Calcium, magnesium, vitamin D, vitamin K, and folate are critical for bone health, immunity, and metabolic functions (46, 47). Additionally, inadequate vitamin D and calcium intake could increase the risk of osteoporosis and related conditions, particularly in later life (46, 47). Folate intake was alarmingly low, with a mean intake of 117.01 μg for males and 121.92 μg for females; compared to the EAR of 400 μg. As a result, 92.3% of males and 94.1% of females consumed inadequate levels of folate. These deficiencies suggest that male and female university students are at risk of long-term health complications like, cardiovascular issues, and compromised immunity (46, 47). Folate is essential for muscle tissue repair, and aids in recovery after exercise (48). This is important in this age group as some males may be engaging in various sports activities. Furthermore, folate also assists in lowering the levels of homocysteine in the blood (49). Therefore it may potentially play a role in reducing the risk of heart disease (48). In summary, micronutrient deficiencies, particularly in calcium, vitamin D, and folate, present serious health risks, especially for women of reproductive age (49). These deficiencies typically reflect a diet pattern low in fruits, vegetables, dairy, nuts, seeds, and healthy fats (monounsaturated and polyunsaturated fats) or a lack of dietary diversity in the students’ diets (50, 51). The high prevalence of inadequate iron intake (79%) and folate inadequacy (92.3%) is concerning since these inadequacies could predispose women of reproductive age to the risk of anaemia and poor pregnancy outcomes (52).

Furthermore, low intake of dietary fiber, coupled with excessive consumption of added sugar among all the respondents, suggests preference for processed and refined foods over whole grains and plant-based sources (53). Inadequate intake of dietary fiber and high consumption of added sugars in the diet of university students suggest a reliance on ultra-processed foods. The high prevalence of nutrient inadequacies observed in this study may be partially attributed to underlying food insecurity among university students. National studies have reported that food insecurity in South African universities ranges from 11% to 38.3% (54), with certain institutions reporting rates exceeding 50% (54). Students from socio-economically disadvantaged backgrounds are particularly vulnerable, and whilst the majority of students are supported by the National Student Financial Aid Scheme (NSFAS) with a monthly food allowance of R1,716 ($97.79), spending is left at a student’s discretion. Whilst many studies have reported that these funds are insufficient (55, 56), concerns have been raised that this allowance may not be consistently prioritized for nutritious food purchases, with some students potentially diverting funds toward non-essential or discretionary expenses, thereby compromising their dietary intake and basic living needs.

Food insecurity not only limits access to nutritious foods but also contributes to dietary patterns dominated by inexpensive, energy-dense, and nutrient-poor foods, which may explain the excessive intake of carbohydrates and protein alongside widespread micronutrient deficiencies identified in this cohort (14). Addressing food and nutrition insecurity is, therefore, essential to improving dietary adequacy and overall student well-being.

To address the burden of nutrient inadequacy and food insecurity, there is a critical need to strengthen food and nutrition literacy among students to enable informed dietary decisions. In parallel, policy mechanisms should be considered to ensure that food allowances are used as intended, specifically for the purchase of nutritious food. This can be achieved through structured food support schemes, such as partnerships with campus cafeterias, local retailers, or food voucher systems to provide affordable, healthy meals. Additionally, implementing digital tracking systems or pre-approved vendor lists can help monitor spending, while collaboration among stakeholders such as universities, student bodies, and policymakers is essential in developing supportive frameworks and guidelines.

These combined efforts could contribute significantly to improving dietary adequacy and long-term health outcomes among university students.

The GDQS effectively captured poor diet quality, with the majority of students classified at high risk for diet-related NCDs. This reflects a broader trend among young adults in developing countries, where processed food consumption is increasing.

Nutrient adequacy and dietary patterns among university students are critical areas of concern, particularly in relation to the GDQS. Recent research indicates that many university students struggle to meet nutrient adequacy due to lifestyle changes, increased stress, and academic demands, leading to poor dietary choices. Many students frequently consume excessive amounts of ultra-processed foods and sugary drinks, which lowers their GDQS scores and causes them to consume insufficient amounts of fiber, vitamins, and minerals (57).

The GDQS is a useful tool for assessing dietary quality by evaluating adherence to recommended food group consumption. Elevated GDQS scores correlate with dietary patterns abundant in fruits, vegetables, whole grains, and lean proteins, which are critical for achieving optimal health outcomes. Nevertheless, a significant number of students do not adhere to these dietary guidelines, with research indicating that merely a limited fraction attains the recommended levels of fruit and vegetable consumption. This dietary inadequacy heightens the risk of nutrient deficiencies and related health issues, such as obesity and metabolic syndrome (58).

Interventions aimed at improving dietary patterns among university students are essential. Recent initiatives, such as campus-wide nutrition education programmes and improved availability of healthy food options, have shown promise in enhancing GDQS scores. These efforts can promote better nutrient adequacy and instill lifelong healthy eating habits (59, 60). Continued research is crucial in clarifying the barriers to healthy dietary habits within this population and formulating tailored strategies that encourage equitable nutrient consumption, thus ultimately reducing the risk of chronic diseases associated with inadequate nutritional quality.

### 4.2 Analysis of students’ dietary patterns and carbon footprint

In this study, it was found that there was a high carbon footprint for both men and women. However, it was much higher for men. The significant gender disparity in carbon footprints aligns with existing literature on meat-based diets. Shifting toward plant-based options could reduce GHG emissions and improve nutritional outcomes.

In recent years, the dietary behaviors of university students have gained significant attention due to their implications for public health and environmental sustainability. Research suggests that university students often adopt diets high in processed foods and low in fruits and vegetables, contributing to poor nutritional outcomes (61). In the demographic cohort aged between 19 and 35 years, dietary selections at the DUT are predominantly shaped by determinants such as time constraints, financial strains, and the availability of convenient and affordable food options within the campus environment. This includes influences from social norms, group dynamics, and the general “student lifestyle” that may prioritize quick and easy meals over more health-conscious food options. This dietary phenomenon not only adversely affects individual health but also exacerbates environmental challenges, given that food production is a major source of GHG emissions. A study by Xu et al. (24), underscores that livestock production is responsible for approximately 60% of the GHG emissions associated with the food sector. Furthermore, a study of university students found that their dietary choices substantially influenced their carbon footprint, with those who consumed more plant-based foods exhibiting fewer emissions. This difference might be fundamentally connected to the observation that males frequently tend to consume a larger portion of food than females. Mitigating climate change requires transitioning to more sustainable eating practices, such as consuming plant-based meals and minimising food waste. Educational initiatives that raise understanding of how dietary decisions affect the environment are key in changing students’ eating habits toward more sustainable diets to promote healthier lifestyles and decrease environmental footprints.

### 4.3 Implications for the university food environment

University food environments influence student food choices. There is a need for targeted interventions to increase access to affordable, nutritious, and sustainable foods on campus. Recent studies concerning the dietary habits of university students have disclosed significant patterns and consequences relevant to health and sustainability. Jakobsdottir et al. (62) suggest that many students opt for convenience-driven diets, often prioritizing fast foods and processed meals over healthier options, leading to inadequate nutritional intake. This pattern causes concern as it not only affects students’ physical and emotional health but also their academic performance and mental well-being (61).

Furthermore, a study by Sahadeo et al. (18) highlighted a growing need for awareness and knowledge among students regarding the environmental impact of their dietary choices. A survey conducted by Mollaei et al. (63) found that students who are aware of sustainable eating are more likely to select plant-based options, substantially reducing their carbon footprint. For instance, individuals adhering to vegetarian or vegan diets can potentially decrease their food-related GHG emissions by up to 50% compared to those who consume meat (64). These findings suggest that universities should undertake strategic initiatives to promote healthier and more sustainable dietary practices. By establishing educational initiatives highlighting the benefits of nutritional diets and environmental sustainability, universities can encourage students to make informed dietary choices. Ultimately, nurturing a culture of mindful eating among students can lead to enhanced health outcomes and contribute to broader sustainability efforts on campus and beyond.

### 4.4 Strengths

The principal researcher and fieldworker, who were trained before the commencement of the data collection process, conducted the 24-h food recall. Respondents were eager to determine their level of knowledge regarding food and nutrition sustainability and what constitutes a healthy diet. The university statistician and supervisory personnel played a pivotal role in guaranteeing the integrity and quality of the data collected for this research study. Validated tools were used in this study, such as the 24-h food recall, which was used to assess dietary adequacy, and the GDQS, which was used to determine the dimensions of diet quality for nutrient adequacy and NCD risk among students at the DUT. A comprehensive dietary toolkit comprising food samples and household measurement instruments was utilized to assist respondents in accurately reporting their portion sizes while conducting the 24-h food recall. The study employed a large sample that was diverse in terms of gender representation.

### 4.5 Limitations

Despite the strengths of this study, it is important to acknowledge the following limitations: The use of self-reported 24-h food recall method may have introduced reporting bias, since respondents may have underreported their portion sizes when describing the quantity and type of foods they consumed. Although food aids were used to assist with portion size identification, the 24-h food recall relies on recent memory and respondents’ ability to recall details about all food eaten over 24 h. It was not possible to calculate the Estimated Energy Requirement (EER), since no body mass index or physical activity level was recorded in this study. The EER was not an objective aligned with this study; hence, it was not considered. However, for future reference, the body mass index and physical activity levels of respondents should be recorded so that the Estimated Energy Requirement can be calculated. The food carbon footprint calculator used to assess respondents’ diet emissions did not contain all food items. Therefore, more research regarding food carbon footprint calculators and databases is required in South Africa to ensure accurate results.

## 5 Conclusion

This study highlights a concerning prevalence of inadequate diet quality and high environmental impact among university students. Most students are at risk for NCDs, and their dietary choices contribute significantly to greenhouse gas emissions. The findings emphasize the necessity of advocating for healthier and more sustainable dietary choices to enhance individual health outcomes while concurrently reducing environmental repercussions. Universities must take a proactive role in creating supportive food environments and educating students about sustainable, healthy eating habits. Addressing these elements can foster improved health outcomes for students and contribute to a more sustainable future.

## Data Availability

The raw data supporting the conclusions of this article will be made available by the authors, without undue reservation.
